# The influence of a signal’s time structure on the perceived noise annoyance of road traffic noise

**DOI:** 10.1007/s40201-021-00655-4

**Published:** 2021-04-13

**Authors:** Jan Felcyn

**Affiliations:** Department of Acoustics, Faculty of Physics, Adam Mickiewicz University, Uniwersytetu Poznańskiego 2, 61-614 Poznań, Poland

**Keywords:** Intermittent noise, Road traffic, Noise annoyance, Time structure, Noise annoyance

## Abstract

**Purpose:**

Road traffic noise is the most common source of noise in modern cities. The noise indicators used to manage noise do not take into account its temporal structure. However, in cities the traffic flow varies during the day, peaking due to congestion and more fluent periods. In this research we sought to analyze how people (giving answers on a numerical ICBEN scale) perceive noise stimuli with the same L_AeqT_ values but different time structures (more/less noise events, different amplitude envelopes).

**Methods:**

31 people with normal hearing took part in an experiment conducted in an anechoic chamber. Participants listened to 18 different noise recordings and rated each of them using the numerical ICBEN scale regarding noise annoyance.

**Results:**

The results showed that only sound level was a statistically significant factor. However, based on people’s remarks about noise, we can also say that the more intermittent the noise is, the more negative feelings it evokes in people.

**Conclusions:**

Time structure does not have a significant influence on people’s judgments about noise annoyance. However, people tend to have a preference for a steady noise rather than an intermittent one.

## Introduction

According to the European Union, air and noise pollution are two crucial factors having a negative impact on the health of people in Europe [1]. Among possibly harmful noise sources, road traffic is the most common. In a recent report, the European Environment Agency pointed out that around 113 million people are affected by long-term road traffic noise whose yearly-averaged sound levels (L_DEN_) are equal to or exceed 55 dBA [2].

There is strong evidence that noise causes many negative health and non-health effects, including cognitive performance [3], sleep disturbance [3], depression and anxiety [4], atrial fibrillation [5], ischemic heart disease [6] etc. There are also some review-type papers summing up all the proven effects of noise, like those by WHO [7, 8], from the ALPNAP project [9], or by group of researchers, e.g. Cai et al. [10]. Based on them, some researchers propose to educate society about noise effects [11]; while the World Health Organization proposed new noise limits [8].

One of the most studied non-health related effects of noise is annoyance, defined by ISO [12] as “one person’s individual adverse reaction to noise”. The common way of studying annoyance is to relate its ratings to the characteristics of noise. Typically, noise is described by some physical parameters, called noise metrics. They are measured over the long-term, averaged over time values, expressed in dBA – e.g. L_AeqT_, L_DEN_, L_N_ [13]. These metrics are used in studies which concern long-term annoyance and are conducted *in situ*, i.e. people in the given area fill in questionnaires regarding their experience of noise in their home. On the other hand, short-term annoyance is studied in laboratory conditions and relates only to the noise heard in the experiment. In that approach, since the stimuli are artificially presented, more noise metrics can be computed, including not only those related to noise intensity/amplitude. In many papers the relation between long-term noise metrics and annoyance assessments was found to be weak, with much variance in the data remaining unexplained [14] or even explained more by non-acoustical factors [15–17]. It suggests that annoyance is a more complex experience, also related to other characteristics of noise or people. Among other interesting factors which have been already investigated are: surface material or vehicle speed [18], having a peaceful room with a quiet façade [19], noise spectrum [20], type of a crossroad [21], different street geometry [22], the presence of green areas [23], personality traits [24], or the number and loudness of noise events/pass-bys [25]. The latter case could be described as time structure and was also analyzed by Kaczmarek and Preis [26], as well as Wunderli [27] and Brink [28]. Kaczmarek and Preis presented four different noise scenarios – characterized by the same L_Aeq,10 m_ values, but with different time patterns. To express differences in the temporal structure, they used a metric called *distortion of informational content* (DR) [29]. It is a fraction in which the sum of the durations of all sounds which exceed the background noise level is divided by the total duration of the stimulus. Other metrics used were psychoacoustical characteristics: roughness and fluctuation strength. Originally introduced by Fastl and Zwicker [30], they reflect fast and slow modulations in sound. This different time pattern was found to be a statistically significant factor influencing annoyance assessments – all the mentioned characteristics were positively correlated with mean annoyance ratings, meaning that intermittent noise was less annoying than a steady one.

Another metric of temporal structure was used by Brink et al. [28]. ‘Intermittency ratio’ (IR) [27] is expressed in percent, between 0 (steady, not-changing noise) and 100 % (loud noise events separated by long silent periods). It was found that IR is a statistically significant factor for both road and railway traffic. For road traffic, when the IR increases, the %HA slightly decreases – suggesting that when the noise metric value is the same, people prefer intermittent noise over its steady type. This conclusion supports the findings from the work of Kaczmarek and Preis.

Regarding psychoacoustical metrics, roughness is a characteristic which can predict the noise annoyance of powered two wheelers [31]. Although the same was found for road traffic noise annoyance by Kaczmarek and Preis, Freitas et al. [18] did not reveal such a relation.

Kaczmarek and Preis presented people with artificially created noise scenarios. There was only one pass-by, multiplied 120 times to create different time patterns. Thus, noise scenarios did not reflect real situations from the environment. Moreover, only one equivalent sound level was observed for all the scenarios, equal to 55 dBA. On the other hand, Brink et al. analyzed IR in a 24-hour period, but for exposure-response curves they used yearly-averaged L_DEN_ indicators. The data about noise annoyance was gathered by questionnaires sent out by post.

Since both studies differ in methodology and the duration of stimuli, we wanted to strictly control this factor in laboratory conditions and ask people to rate short-term annoyance caused by different road traffic noise stimuli. These stimuli were created directly from field recordings of a real city traffic flow. We assume that time structure should be a significant factor regarding road traffic noise annoyance, and that at least one metric should correlate with its ratings.

## Method

### Stimuli

All the stimuli used in this study were created from recordings of road traffic noise made in the field. Thus, all stimuli consisted of real vehicles’ pass-bys recorded in everyday traffic. In this part firstly we characterize recordings, then we discuss the process of the construction of stimuli, and finally we present the method for their calibration and representation.

#### Recordings

The recordings of road traffic noise were made in Poznań, a city in Poland with ~ 500 000 inhabitants. They were made using an ambisonic microphone, SoundField ST450 paired with a Head Acoustics Squadriga II recorder. At the output, we got B-format recordings (4 channels, one omnidirectional and three 8-shaped). More details on ambisony can be found in [32]. The recordings were made 10 m from the middle of a four-lane road and at a height of 1.2 m.

The recordings capture the natural flow of the traffic. Thus, it was not single pass-bys that were recorded, but whole ‘packages’, i.e. all the vehicles which established a consistent group, mainly controlled by traffic lights in the intersections nearby. As the flow varies in time, some packages contain only a few pass-bys while others have dozens of them and last more than 2 min. In every package, light and heavy vehicles were counted and their average speed was estimated.

#### Construction of stimuli

On the basis of information about road traffic intensity (a four-lane road, data from inductive loops near intersections) we created 10-minute noise stimuli with the number of light and heavy vehicles corresponding to the common situation. To obtain such scenarios, we selected the best (in terms of quality) road traffic recordings and used them in the process of constructing the stimuli. Ten different recordings were chosen, with 331 light vehicles and 20 heavy ones. The recordings lasted about 590 s altogether. The construction of stimuli was done randomly, i.e. the order of ten road traffic packages was pooled.

**Fig. 1:**
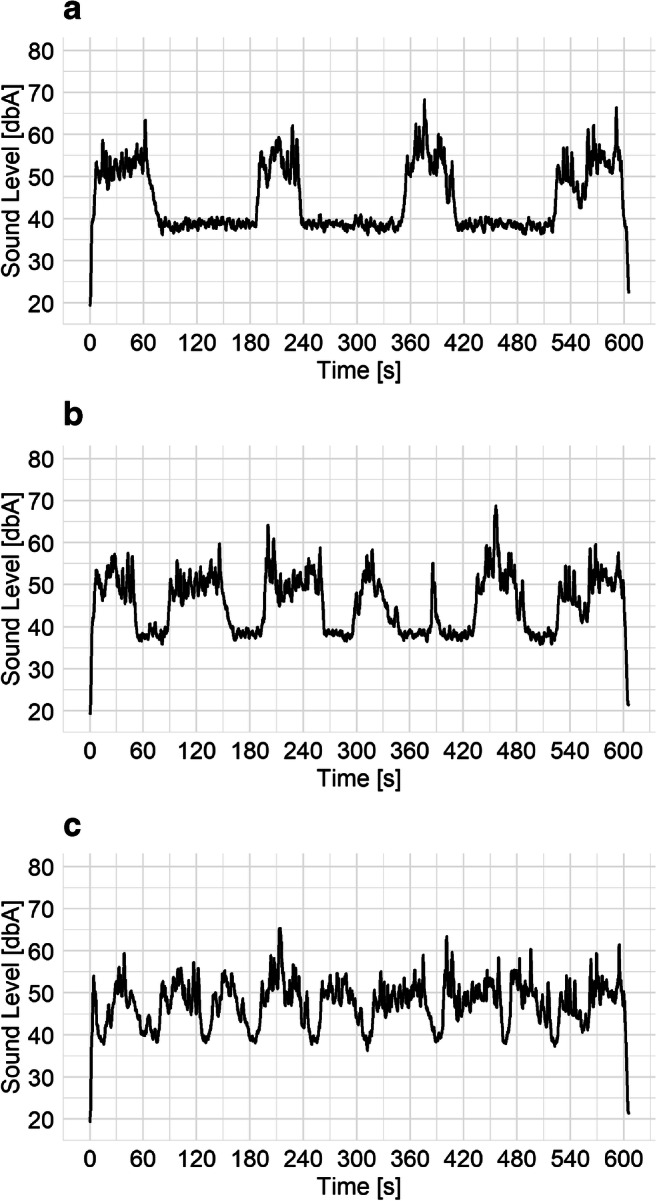
Changes of sound level values in time for three different proportions (**a** − 50 %, **b** − 75 %, **c** − 100 %). All stimuli had the same 10-minute equivalent sound level of 50 dBA

Based on the approach from Kaczmarek and Preis [26] and traffic lights in the city, we wanted to simulate different traffic flows. Thus, three different flows were represented. Namely, 100 %, 75 and 50 % (the factor labelled as ‘proportion’). Those numbers represent the situation when x % of the whole duration of the stimulus is busy with road traffic and the rest is just background noise (continuous, steady traffic flow versus intermittent/pulsating flow). All three examples are presented in Fig. 1.

Each proportion was also set to represent three different 10-minute equivalent sound levels. Those levels were 50, 55 and 60 dBA. Of course, this approach is very limited, but having so few values was necessary to keep the experiment reasonably short and manageable, without long-lasting experimental procedures. We also added some background noise to all the stimuli, the same as that which was used in [26]. It was quasi-stationary noise recorded at a large distance from a city road infrastructure. For all the stimuli, volume manipulation was performed to keep the overall road traffic L_Aeq,10 m_ = 60, 55, or 50 dBA and the background level at around 40 dBA.

Volume manipulation requires more detailed explanation. We have already mentioned that each stimulus consisted of several road traffic recordings made in the field. Thus, each recording had different sound levels. It can be seen from Fig. 1a that the maximum sound level of the third package is clearly higher than of the second one. This means that each package had a different sound level, but the value of L_Aeq,10 m_ for each stimulus was set to 50, 55 or 60 dBA.

On the other hand, we wondered how the perception of noise could change when all the packages were artificially set to have the same sound level. In this approach, every road traffic package was set to have the same sound level value – i.e. 50, 55 or 60 dBA. This means that, e.g., for a 50 % proportion noise scenario presented at 50 dBA, all four sound packages were changed in volume to emit a sound level of 50 dBA each. Obviously, such a modification influences the overall sound level, i.e. L_Aeq,10 m_. For 50 and 75 % proportion cases, L_Aeq,10 m_ values were slightly lower in this case than in the previous one (see Table 1 for more details).

In general, keeping all the recordings in the stimulus at the same sound level reflects the stability of short-term sound level values, while keeping them untouched mirrors some fluctuations in these values. Finally, it is necessary to mention that in noise maps the values of noise indicators are given in 5-decibel intervals, i.e. their values are represented as 45–50 dBA, 50–55 dBA etc. In our case, both methods produced stimuli which could be represented in such groups. Thus, from the ‘noise mapping’ point of view, all were the same (from the same L_Aeq,10 m_ range).

**Fig. 2:**
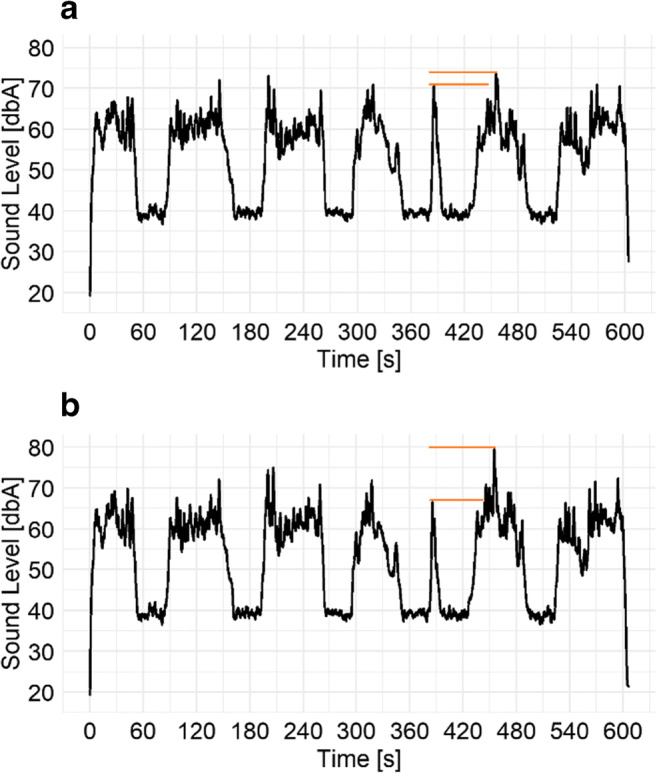
Two stimuli with different method of setting the sound level. **a **events, where each recording was manipulated to give the same equivalent sound level of 60 dBA; **b **scenario, where original differences in amplitude between recordings were preserved. The orange horizontal lines depict the difference in maximum sound level between two neighbouring sound packages

In the following parts of this text, the case where all the sound level proportions of the sound packages have been preserved will be called a ‘scenario’. The case where all the sound packages were adjusted to give the same sound level values will be referred to as ‘events’. An example of the difference between ‘scenario’ and ‘events’ for the same proportion (75 %) is presented in Fig. 2.

In summary, noise scenarios with different time structures have been developed through two methods of manipulation. In the following sections we will refer to them using the variable ‘time pattern’ (for manipulation between packages, i.e. ‘scenario’ or ‘events’ situations) and ‘proportion’ (for manipulation of the time when busy traffic was presented, i.e. 50 %, 75 % and 100 %).

**Fig. 3:**
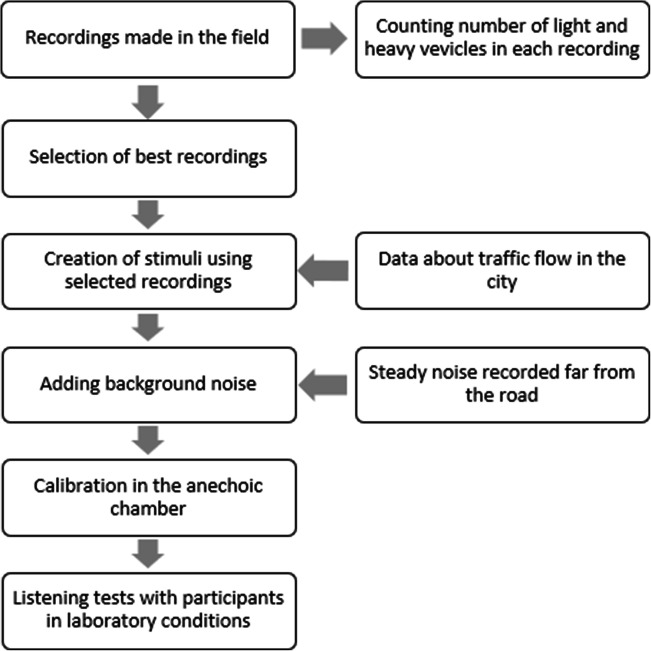
All the steps made during the creation of stimuli with road traffic noise from original recordings

To sum up, we prepared 18 different road traffic noise situations (each lasting 10 min): 3 various proportions x 3 various sound levels ranges x 2 different time patterns. All stimuli were based on B-Format (four channels). The output was obtained using a custom-designed program, written in C#, which transformed B-format recordings into 26-channel .wav files. To limit sudden changes in the amplitude envelope at the beginning and the end of stimuli, they were faded in and out, with 2 s linear fades. The overall sequence of steps in the creation of stimuli is presented in Fig. 3.

#### Calibration and representation

**Fig. 4:**
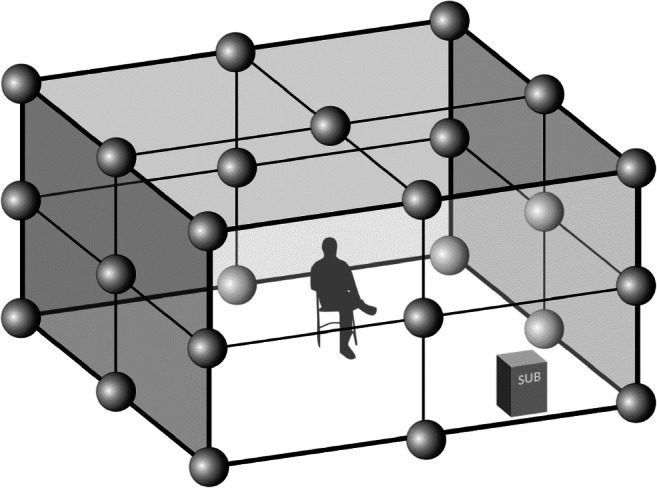
An ambisonic configuration in an anechoic chamber made with 25 Yamaha HS5M loudspeakers. Channel 26 is a sub-bass

The experiment was conducted in the anechoic chamber located at Chair of Acoustics, Faculty of Physics, Adam Mickiewicz University in Poznań, Poland. We used a 1st order ambisony which was preserved with 26 loudspeakers (1 was a sub-bass). The configuration can be found in Fig. 4.

As we have mentioned earlier, 18 noise stimuli were created and presented to the participants. For each participant they were presented in a different (random) way. All the cases and their characteristics are presented in Table 1.

**Table 1:** Basic characteristics of all noise stimuli presented in the experiment

Scenario	Road traffic proportion	Road traffic sound level range (L_Aeq,T_)	Time pattern	L_AEq,10 m_ [dBA]
1	0.50	45–50	events	47.1
2	0.50	45–50	scenario	50.0
3	0.50	50–55	events	51.8
4	0.50	50–55	scenario	55.0
5	0.50	55–60	events	56.9
6	0.50	55–60	scenario	60.0
7	0.75	45–50	events	48.5
8	0.75	45–50	scenario	50.0
9	0.75	50–55	events	53.9
10	0.75	50–55	scenario	55.0
11	0.75	55–60	events	58.1
12	0.75	55–60	scenario	60.0
13	1.00	45–50	events	50.0
14	1.00	45–50	scenario	50.0
15	1.00	50–55	events	55.0
16	1.00	50–55	scenario	55.0
17	1.00	55–60	events	60.0
18	1.00	60	scenario	60.0

A comfortable chair was placed in the middle of the lower level of the chamber. Every listener that took part in the experiments sat in this chair. In the sitting position, the head of the participant was about 1.2 m above the ground level. At the same height we placed a calibrated SVANTEK SV11 microphone, which was connected to a SVAN 945 A sound meter placed outside the chamber.

That point was used as the zero point of 3D Cartesian coordinates. The positions of all the other loudspeakers were measured and described in the same frame of reference. Then, all these coordinates were put into a custom-designed program, written in C#, which transformed B-format recordings into 26-channel .wav files. Compensation of delays (resulting from different distances between the zero point and each loudspeaker) was also applied.

This compensation was also used to calibrate the entire system. First, all Yamaha HS10 speakers were set to the same value using the volume control knob. Secondly, we created an audio session in Samplitude Pro X DAW. 26 different channels with a 10-minute pink noise sample were set. Then, 26 additional buses were created, each representing one speaker. Each track with a pink noise was then connected to the corresponding bus. Next, a noise was emitted from every loudspeaker separately and, using a fader of a corresponding bus in the audio session, the volume was changed to give the same sound level from every speaker (L_Aeq,1 m_ = 75 dBA). The sound level was measured 5 times in 1-minute periods for every loudspeaker. Then each stimulus was also measured to give the appropriate equivalent sound level.

For the purpose of objective analysis of each stimulus in the way that it was perceived by participants, each stimulus was recorded with a dummy head. We put it on a microphone stand and then installed it on a chair – to simulate the participants’ situation when sitting. We used a Neumann KU100 dummy head. The head was placed on the armchair used by the participants, at the same height. Finally, from such recordings all the required metrics (sound level, loudness, roughness, fluctuation strength, IR and DR) were computed, either by hand or using the psychoachoustical software ArtemiS. Loudness, roughness and fluctuation strength were also computed as percentile statistics, i.e. for 5 %, 10 %, 20 %, 30 %, 40 %, 50 %, 60 %, 70 %, 80 %, 90 and 95 %. In this case, percentiles express the value of a given metric which is reached or exceeded in x% of the measurement time. For example, loudness 5 % represents a value which can be observed in the stimulus during 5 % of its entire duration.

### Participants

The participants were recruited through an announcement at the faculty, as well as information published on social media. They were checked to see if they had normal hearing in accordance with the WHO 1997 standard (not more than 25 dB HL for 0.5, 1, 2 and 4 kHz) and all of them met these requirements. 31 participants took part: 19 women and 12 men (the mean age was 28.2 with SD of 7.4 years), and they were paid for their participation.

### Procedure

All the participants were asked to fill in a short survey about their feelings and attitudes towards different noise sources. The survey used in this procedure was created based on a short version of the NoiSeq survey [33]; it also comprised some aspects previously mentioned in the literature [14, 34–37]. However, the results of these questionnaires will not be analyzed in this paper.

Only a single annoyance question was posed, based on guidelines made by Fields and al. [38], in Polish [39]. The content of the question could be translated as follows:

Thinking about the last recording, please rate on the numerical scale from 0 to 10 how much the noise source disturbed/irritated or annoyed you. If the noise was not annoying at all, please choose 0, if it was extremely annoying, choose 10, if the experience was ‘between’, choose the number between 0 and 10. Which number, from 0 to 10, best describes how disturbing/irritating/annoying the noise was?

We used a numerical ICBEN scale (from 0 – not annoying at all to 10 – extremely annoying). There was also one open-field question in the questionnaire, which allowed participants to provide *‘any other interesting thing/detail you noticed in the recording’*.

Participants were asked to bring a book and read it during the experiment, and to refrain from analyzing the details of the presented recordings. Using a talkback channel between the chamber and the control room, after each stimulus the participant was informed about the end of the recording and asked to fill in the survey (in the paper form). When the participant declared him/herself ready for the next stimulus, another noise scenario was started, and the participant was asked to continue reading. Two sessions were carried out (on separate days) and in the middle of each session a short break was made for participants to have a rest.

## Results

### Analysis of respondents’ reliability and the normality of their annoyance ratings

Two tests were applied to the data to find out how consistent our respondents were in their answers: Cronbach’s alpha and intra-class correlation (ICC) – not only for all participants, but also for the case ‘when an item dropped’. The results of these tests revealed that one participant, ‘JL’ negatively correlated with the other listeners. Thus, ‘JL’ was excluded from further analyses. This means that the results of 30 participants were taken into account.

Testing the normality of the annoyance ratings was done using the Liliefors test. The assumptions were not met.

### Proportion, time pattern, sound level range and their influence on road traffic noise annoyance ratings

As has already been mentioned, road traffic noise stimuli were constructed in such a way that L_Aeq,10 m_ was constant, while the time structure of the whole recording varied. This variation was made in two ways: by manipulating the equivalent sound levels of each package in a recording or by manipulating the proportions between ‘quiet’ periods (when only background noise was present) and ‘busy’ periods (when car pass-bys were presented). The first manipulation in our analyses is called ‘time pattern’, while the second is named ‘proportion’.

Because, as was said before, the assumption of normality was not met, we took all the respondents’ ratings of all 18 road traffic stimuli and put them into a robust analysis of variance from a ‘walrus’ package in R. ‘Robust’ in this approach means ANOVA computed for trimmed means – as it was impossible for a three-way design to use the bootstrap method or medians. The three independent variables analyzed in this section were: 5dB sound level range, time pattern and proportion. The results of the analysis can be found in Table 2.

**Table 2:** The results of the robust ANOVA analysis with noise annoyance ratings as the dependent variable and cars’ sound level, time pattern and proportion as factors

Factors	Q	p
Proportion	0.6792	0.7200
Sound Level range	46.4578	0.0001
Time pattern	3.2949	0.0710
Proportion: Sound Level range	1.4549	0.8370
Proportion: Time pattern	1.4777	0.4800
Sound Level range: Time pattern	0.6558	0.7220
Proportion: Sound Level range: Time pattern	0.7456	0.9470

Q is the robust analog of the F coefficient, p is the statistical significance

As can be seen from Table 2, statistical significance was observed only for the road traffic sound level range, and not for the proportion of cars, nor for time pattern. Moreover, none of the interactions were considered statistically significant. Post hoc analysis revealed that the sound level range was statistically significant for all possible combinations (with p around 0.001 and 0.005).

**Fig. 5:**
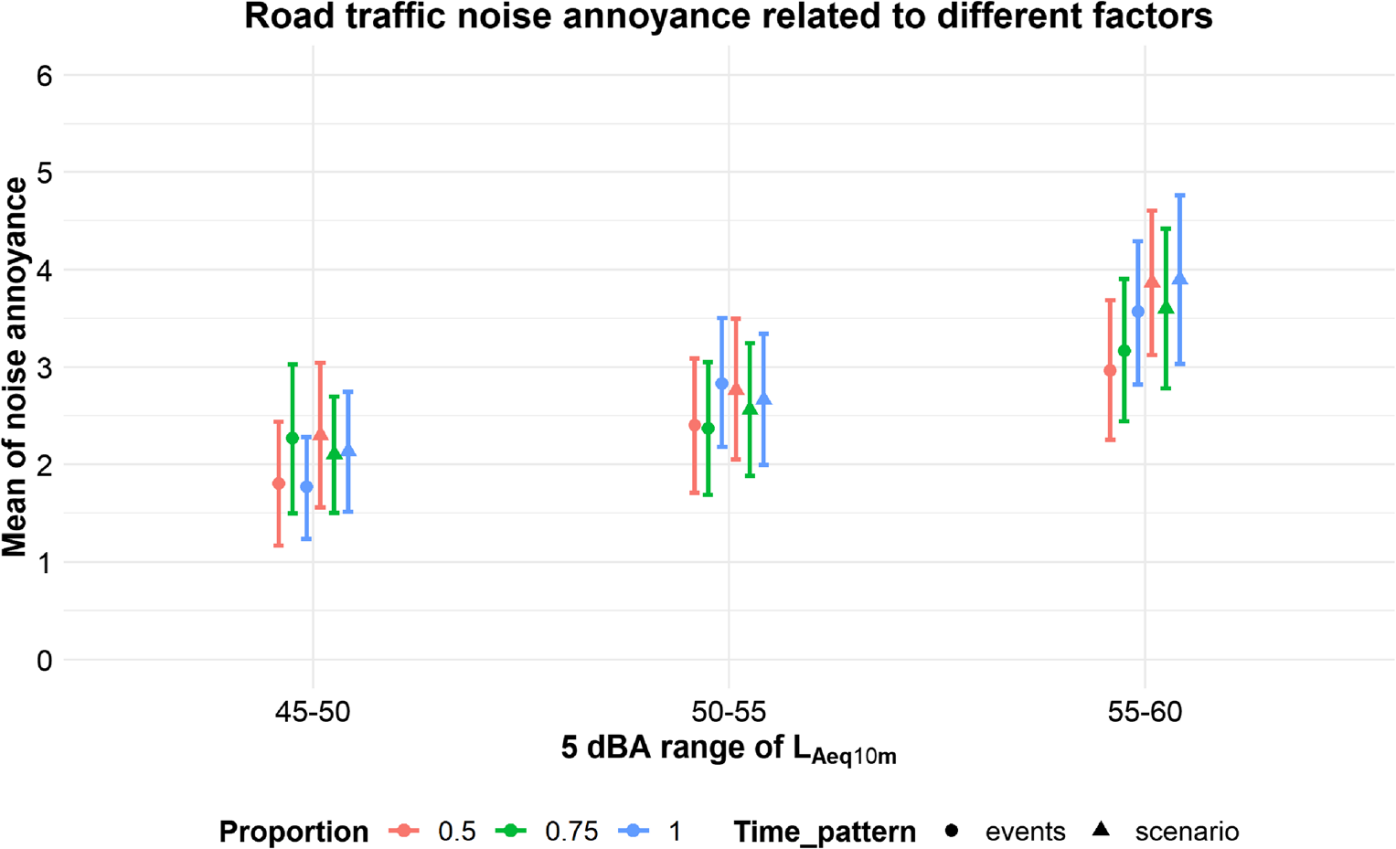
Road traffic noise annoyance related to different factors. Bootstrapped means with 95 % confidence intervals are shown for different time patterns and different proportions of cars

More suggestive information is presented in Fig. 5. Higher sound levels imply higher means of noise annoyance. However, there is one more interesting thing to note: in almost all cases (except two: a sound level of 45–50 dBA with 75 % proportion of cars, and 50–55 dBA with 100 % proportion of cars), people gave higher ratings to globally averaged stimuli (‘scenario’, triangles) than to cases with each package set separately (‘events’, circles). Nevertheless, from the statistical point of view it seems that only the sound level range has a significant impact on people’s judgments about the annoyance caused by road traffic noise.

### IR, DR and psychoacoustical characteristics

The second step of the analysis was to find out if any of the given metrics correlated with mean annoyance ratings.

**Fig. 6:**
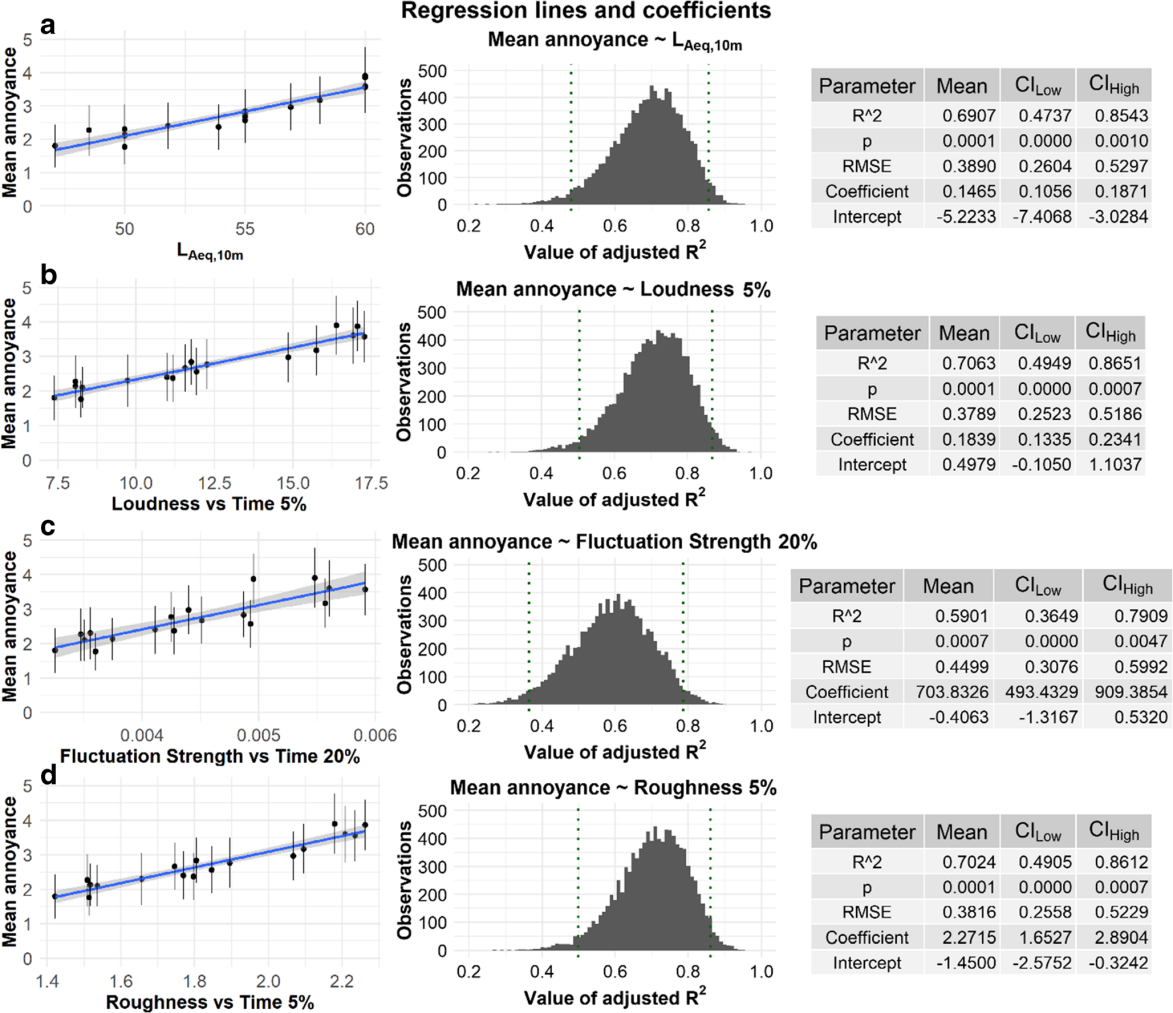
Four simple linear regressions established between mean annoyance ratings and various sound metrics: **a ** L_Aeq,10 m_, **b **Loudness 5 %, **c **Fluctuation Strength 20 % and **d **Roughness 5 %

At first, the overall analysis of a linear regression between mean annoyance ratings and noise metrics revealed that both psychoacoustical characteristics (fluctuation strength and roughness) are good predictors of road traffic noise annoyance. The best fit was found for roughness 5 % and fluctuation strength 20 %. However, statistical significance was found neither for IR nor for DR metrics. Linear regression was also established for the relation between mean annoyance ratings and loudness 5 % as well as L_Aeq,10 m_. Each regression was computed in a bootstrap procedure (with 10,000 replications) and all assumptions were checked and met. Thus, four simple linear regressions are shown in Fig. 6. An attempt was also made to use several metrics to establish multiple linear regression, but no combination was found to be statistically significant.

Analyses were also made for three separate dynamic ranges (i.e. 45–50, 50–55 and 55–60 dBA). In this case some statistically significant results were found only in the group 55–60 dBA. Linear regression was established between mean annoyance ratings and L_Aeq,10 m_ (R^2^ = 0.83, p = .0076) and Roughness 5 % (R^2^ = 0.67, p = .0288). However, that subgroup consisted of only 6 stimuli, 4 of which had the same L_Aeq,10 m_ = 60 dBA. Thus, the relation with the equivalent sound level is rejected because of the obvious bias in the results.

### Positive and negative feelings about presented noise

In addition to the analysis of relations between mean annoyance ratings and noise metrics values, we decided to analyze the answers given to the open question in the questionnaire. In this question we asked them to make some comments about their feelings, emotions or perceptions, if they felt they were important. As it was an open question, we did not receive many comments (70 out of 540 stimuli presentations, giving ~ 13 %). However, we manually segregated them into some groups and then those groups were classified in a way to represent either a positive or negative attitude towards a given noise scenario. Both groups are described below:

- positive attitude: ‘almost inaudible’, ‘far from the road’, ‘mainly peaceful’, ‘makes me sleepy’, ‘no annoying sources’, ‘peaceful traffic flow’.
- negative attitude: ‘annoying steady noise’, ‘music from a car’, ‘heavy vehicles’, ‘impossible to read’, ‘disturbing sudden noise’, ‘many different noise levels’, ‘road traffic intensity was high’, ‘speeding cars’.

It has to be mentioned that one recorded car had loud music turned on, so it was possible to notice it. Another thing is that when the proportion was not 1, people had two opposite attitudes towards the fragments with background noise. Several respondents tended to describe them as peaceful or like a distant road, while the others said that it was an annoying steady noise.

Thus, as two different attitudes could be observed, we decided to run a simple t test using this limited data (only observations with comments assigned to one of either groups) to find out if there is any statistically significant difference. Because the groups were not equinumerous (49 negative comments and 21 positive), we used a *t1waybt()* function from the ‘WRS2’ package in R which enables a bootstrapped version of t-test to be computed. 10,000 replications were used.

The results showed a clear difference between both groups in relation to the values of noise annoyance ratings: t = 33.87, p < .0001, variance explained at about 63 %, and an effect size of 0.79. As the mean value of annoyance assessment for positive comments was 1.62 and for negative ones 4.04, it is clear that people with a negative attitude gave higher values of annoyance than those with positive feelings.

**Fig. 7:**
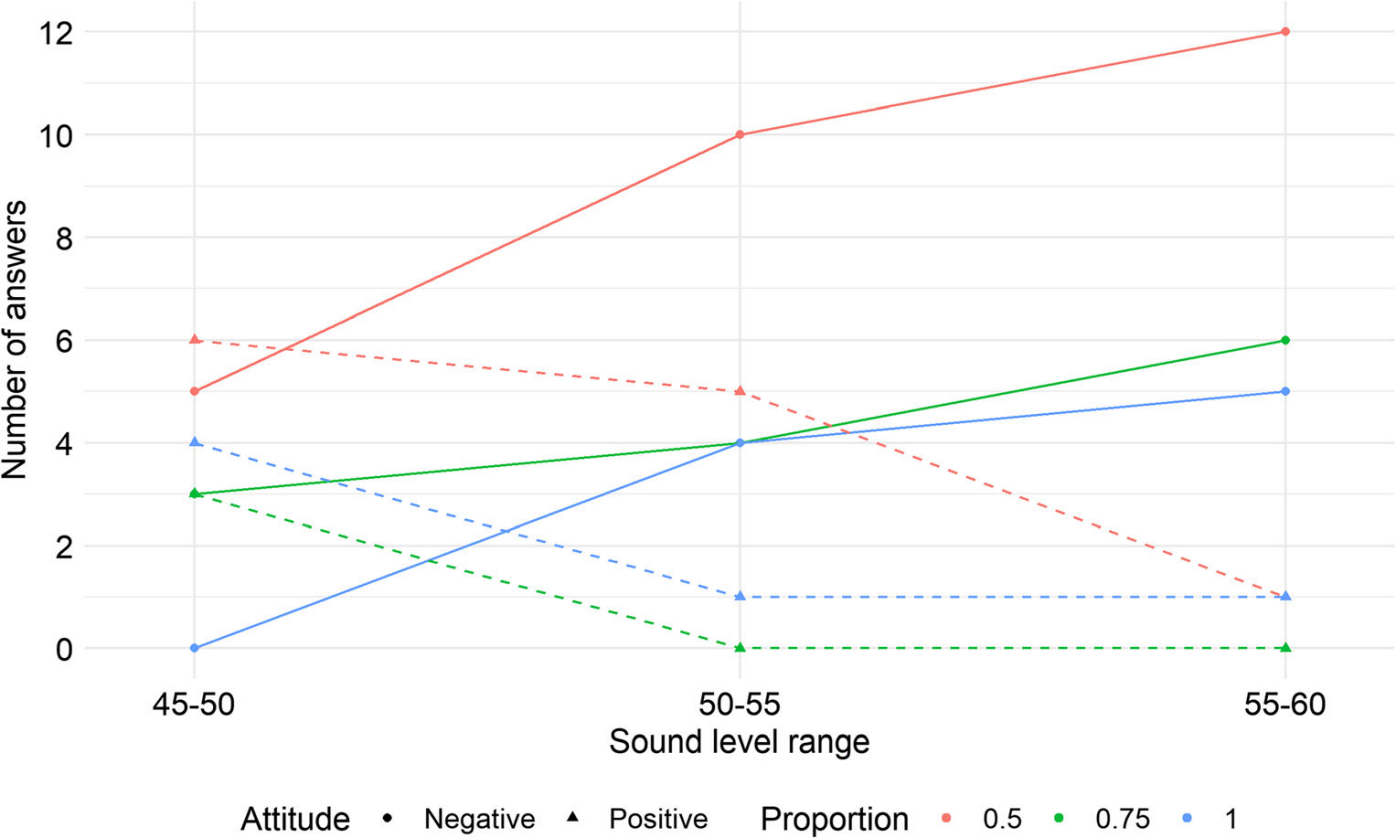
The number of positive (dashed line) and negative (solid line) comments about different noise scenarios in relation to road traffic sound level range and the proportion between road traffic noise and background noise

The number of negative and positive comments related to different proportions and the sound level values are presented in Fig. 7.

## Analysis

### Different sound metrics and their influence on noise annoyance ratings

In this experiment, one of our goals was to find out whether different proportions between background noise and road traffic, while keeping the same L_AEq,10 m_, or different time pattern variables, have an impact on people’s judgments of noise annoyance. The results showed that four different metrics has a significant influence: L_Aeq,10 m_ (with adjusted R^2^ = 0.69), loudness 5 % (R^2^ = 0.71), fluctuation strength 20 % (R^2^ = 0.59) and roughness 5 % (R^2^ = 0.70). The first two parameters are related to sound intensity (and loudness is also related to its spectral nature) while the latter two reflect both slow and fast modulation – thus, temporal structure. The temporal structure of sound and its composition of loud and silent periods is also described by IR and DR. However, neither of them was found to be statistically significant in this study.

Although L_AEq,10 m_ accounts for almost 70 % of the variance explained in people’s answers, it is necessary to mention that this relation is biased. There are several recordings for which L_AEq,10 m_ are the same, meaning that for some stimuli the L_AEq,10 m_ value remains the same but annoyance ratings differ. This can be clearly seen in the top part of Fig. 6. Thus, this relation should be analyzed with particular care.

Nevertheless, we want to discuss the high percentage of variance explained both by L_Aeq10m_ and loudness. In this case, we conducted our experiment in laboratory conditions. Thanks to this, many factors could be controlled, limiting their possible influence on the participants’ judgment. Moreover, the participants rated only one type of noise (road traffic), so there was no potential influence from other sources. In this context, the high correlation between mean annoyance ratings and sound level values is not very surprising.

Regarding the temporal structure of sound, both fluctuation strength 20 % and roughness 5 % were found to be good predictors, although roughness had a clearly better adjusted R^2^ value. It seems that in perception the most important role is played by those moments when the sound differs significantly from its ‘common’ structure. Especially for roughness, it is clear that the overall perception of a 10-minute stimulus depends mainly on the 30-second ‘roughest’ period (5 % of the whole duration). The same can be said about loudness and the loudest parts of the stimulus. Thus, it seems reasonable to conclude that the overall perception depends on some abruptions and deviations from the ‘normal’ traffic flow.

Interestingly enough, this phenomenon was not revealed by IR or DR – as they were not found to be statistically significant factors. However, in this research neither metric achieved extreme values. The range of DR was between 31 and 92 %, while IR had values between 43 and 75 %. Perhaps higher ranges of both of them could reveal some tendencies – as was the case for example in [28].

Neither of the factors seen in Fig. 5 were found to be statistically significant. For the time pattern variable, generally all the cases when a whole stimulus was adjusted to a given sound level (‘scenario’) are rated higher (with two exceptions) than those in which every road traffic package (‘events’) was adjusted separately. This should not be surprising, as when e.g. there are 5 different road traffic packages, each with the same sound level of 60 dBA and the rest of the time in the stimulus is filled with a background noise at 40 dBA, globally the equivalent sound level measured for 10 min would be lower than 60 dBA.

Moreover, there are also no statistical differences between various noise stimuli which are classified within one of three possible 5-decibel intervals (i.e. 45–50, 50–55 or 55–60 dBA). It seems that these intervals, used in the noise maps of a given area, are justified, and it is not necessary to provide more detailed information, as this does not impact people’s noise annoyance ratings.

Another fact is that a rising proportion (from 0.5 to 1) between road traffic noise and background noise does not always relate to the rising tendency of noise annoyance ratings. For lower sound level ranges (45–50 and 50–55 dBA) we can observe something like a bias – mean ratings oscillate around the same value with small differences, sometimes even giving highest ratings for the proportion of 0.5, not for 1. The situation becomes more ‘organized’ for the highest sound level of 55–60 dBA. In this case, the 0.5 proportion has a lower rating than 0.75 and 0.75 has an assessment lower than 1. Of course, those tendencies or differences are not so large as to have any statistical significance. However, for this subgroup a linear regression was established between mean annoyance ratings and roughness. It seems that for louder stimuli roughness plays more important role than for softer ones. It may be that the amplitude modulation in such stimuli is high enough to be an important factor influencing people’s judgments. It is probably just as important as sound level, but because of the already mentioned bias, we do not take this result to be certain.

### Positive and negative attitudes towards road traffic stimuli

From Fig. 7 it can be seen that if the sound level increases, more negative comments occur and there are fewer positive ones. On the other hand, the higher the proportion, the fewer negative comments there are. This is probably related to the problem of intermittent noise, it is easier to adapt to loud but steady noise, than to a situation where some differences in sound levels occur. In our opinion, this is also related to Fig. 5. When sound level ranges are low (45–50, 50–55 dBA) people are mixed in their opinions, but when sound level is high enough, the proportion (or rather roughness) starts to influence their perception, and this is also reflected in Fig. 5.

It is obvious that negative comments should be more frequent when the sound level increases. Thus, in our opinion, the information from Fig. 7 is interesting mainly regarding the number of positive/negative comments related to the proportions between road traffic noise and background noise. This shows that people tend to have a more negative attitude towards noise sources when they are presented in different ‘noise packages’ separated by ‘quiet’ periods (intermittent noise). Nevertheless, this tendency was not statistically significant (as has been shown in the ANOVA results in Table 2), nor was it reflected in the relations between annoyance ratings and DR or IR values.

## Conclusions

The comparison of the results obtained in this research and in the work of Kaczmarek et al. [26] presents some ambiguity. Those authors found that the proportion (expressed with DR) was statistically significant, while we did not. Possible reasons for this can be discussed. Firstly, the authors presented all their stimuli with the same L_Aeq,T_ value, so the sound level was not a factor in their work. In our research, we provided three different sound levels – it is possible that this factor was the most important one and ‘covered’ the influence of the other variables. Finally, Kaczmarek et al. used artificially prepared noise scenarios, while we focused on real environmental conditions. This means that our stimuli were constructed from road traffic packages, not single pass-bys. This constant flow of road traffic may be a reason why there are no differences between various proportions and time patterns.

Comparing our results with those obtained by [28] we can say that our results do not support their findings. But we have to bear in mind that the authors analyzed long-term annoyance [37, 40], i.e. they asked people to rate their general feeling about noise annoyance in in situ research. In this paper we describe a laboratory approach, and thus analyzed short-term annoyance. This may be the main reason why our findings differ. Moreover, the IR values range was limited; possibly their higher differentiation would affect people’s judgments.

The collected data and the analyses calculated on the basis of it led to many interesting conclusions and relations.

1. Although some tendencies can be observed, no statistical significance of time pattern or proportion was found. Neither IR nor DR were found to be statistically significant.
2. Annoyance ratings correlate well with the equivalent sound level and loudness – which depicts sound intensity. Linear regressions were also established for roughness 5 % and fluctuation strength 20 %, which relates to fast and slow amplitude modulation in sound.
3. The 5dB intervals used in noise maps seems to be enough to reliably show data, as no statistically significant differences were found within 5dB subgroups. However, for the loudest one (55-60dBA) roughness was found to be a good predictor of noise annoyance ratings.
4. People’s negative reactions to noise increases with an increase in sound level values but decreases with an increase in the proportion factor value. This could suggest that people prefer continuous noise over intermittent noise.
5. When people report their positive or negative feelings about the perceived noise, there is a statistically significant difference between mean annoyance ratings for both groups. People with positive feelings give lower annoyance ratings than those whose reactions are negative. This finding could be a basis for noise policies – e.g. if it is impossible to reduce noise levels, at least one could convince people to have positive feelings about the sound source.

## Data Availability

Not applicable.
